# The Expression of Type-1 and Type-2 Nitric Oxide Synthase in Selected Tissues of the Gastrointestinal Tract during Mixed Mycotoxicosis

**DOI:** 10.3390/toxins5112281

**Published:** 2013-11-22

**Authors:** Magdalena Gajęcka, Ewa Stopa, Michał Tarasiuk, Łukasz Zielonka, Maciej Gajęcki

**Affiliations:** 1 Department of Veterinary Prevention and Feed Hygiene, Faculty of Veterinary Medicine, University of Warmia and Mazury in Olsztyn, ul. Oczapowskiego 13/29, Olsztyn 10-718, Poland; E-Mails: lukaszz@uwm.edu.pl (L.Z.); gajecki@uwm.edu.pl (M.G.); 2 Veterinary Clinic, Ewa Stopa DVM, ul. Dąbrowskiego 15, Iława 14-200, Poland; E-Mail: ewastopa@autograf.pl; 3 BIOMIN Polska Sp. z o.o., ul. Grochowska 16, Warszawa 04-217, Poland; E-Mail: michal.tarasiuk@biomn.net

**Keywords:** mycotoxins, low doses, zearalenone, deoxynivalenol, gene expression, nitric oxide synthases

## Abstract

The aim of the study was to verify the hypothesis that intoxication with low doses of mycotoxins leads to changes in the *m*RNA expression levels of nitric oxide synthase-1 and nitric oxide synthase-2 genes in tissues of the gastrointestinal tract and the liver. The experiment involved four groups of immature gilts (with body weight of up to 25 kg) which were orally administered zearalenone in a daily dose of 40 μg/kg BW (group Z, *n* = 18), deoxynivalenol at 12 μg/kg BW (group D, *n* = 18), zearalenone and deoxynivalenol (group M, *n* = 18) or placebo (group C, *n* = 21) over a period of 42 days. The lowest *m*RNA expression levels of nitric oxide synthase-1 and nitric oxide synthase-2 genes were noted in the sixth week of the study, in particular in group M. Our results suggest that the presence of low mycotoxin doses in feed slows down the *m*RNA expression of both nitric oxide synthase isomers, which probably lowers the concentrations of nitric oxide, a common precursor of inflammation.

## 1. Introduction

The liver and intestinal mucosa are subjected to high antigen loads and highly varied antigen molecules ingested with feed. Foodborne microorganisms colonize the digestive tract [1]. The intestinal barrier function involves a series of mechanisms which control absorption through the mucous membrane and prevent intestines against harmful substances. In a stable physiological state, small numbers of antigens are transported across the intestinal barrier which can quickly identify and eliminate foreign intruders that pose a health risk. Two types of immune responses are involved in this process: innate (nonspecific) immunity which is the body’s first line of defense against infections and adaptive (specific) immunity which is further subdivided into cellular and humoral immunity. If the intestinal barrier function is disrupted by harmful substances ingested with food, such as mycotoxins [2,3], other antigen molecules or microorganisms, the structural continuity of the mucous membrane may be disrupted and the number of signaling molecules may be altered, leading to various pathological states. In a normal host, equilibrium is restored when the pathological factor is eliminated, but in susceptible hosts, intestinal permeability may be increased which, in extreme cases, can lead to chronic inflammations [4]. 

Nitric oxide (NO), first described as an endothelium-derived relaxing factor (EDRF), plays various roles in basic life functions of an organism. NO is one of the smallest known biologically active molecules. In stable physiological states, it acts as an intracellular signaling molecule, and at higher concentrations, it plays the role of an autocrine and paracrine molecule which regulates the immune response [5] or a factor which controls the peristalsis of the entire gastrointestinal tract [6]. NO levels are determined by the activity of various nitric oxide synthase (NOS) isomers [7,8]. Three basic forms of NOS have been identified to date: NOS-1, NOS-2 and NOS-3. They were previously classified as: NOS-1–nNOS (neuronal NOS) or NOS-I; NOS-2–iNOS (inducible NOS) or NOS-II; and NOS-3–eNOS (endothelial NOS) or NOS-III [6]. Mammalian cells contain two constitutive NOS enzymes—NOS-1 and NOS-3 which synthesize NO in response to an increase in calcium ion (Ca^2+^) concentrations inside a cell [9]. In some cases, those enzymes can be activated independently in response to stress. 

The activity of the inducible isoform of NOS-2 is not determined by intracellular concentrations of Ca^2+^, but by calmodulin binding. Higher Ca^2+^ concentrations inside a cell increase calmodulin levels and supports calmodulin binding with NOS-1 and NOS-3, leading to momentary intensification of NO synthesis [10]. Unlike isoforms NOS-1 and NOS-3, NOS-2 can bind with calmodulin even at low intracellular concentrations of Ca^2+^. NOS-2 activity is not affected by changes in Ca^2+^ levels, which prolongs synthesis [11] and increases local NO concentrations in the cell in comparison with the remaining NOS isoforms [12,13,14]. 

NOS-2 is found mainly in the central nervous system, peripheral nervous system, skeletal muscles, pancreatic islets, endometrium and the macula densa. NOS-2 modulates the transmission of neural signals, regulates nephron functions and controls gastrointestinal peristalsis [6]. NO produced by NOS-2 also acts as a neurotransmitter, in particular in the non-adrenergic non-cholinergic (NANC) nervous system. NOS-1 is activated in the first hours after cytokine release. Successive periods are characterized by a predominance of NOS-2 which synthesizes significant amounts of NO [15]. 

NOS-2 is also observed in macrophages, cardiac muscle, liver, smooth muscles and vascular endothelium where it is synthesized in response to endogenous and exogenous agents such as bacterial lipopolysaccharides, pro-inflammatory cytokines (IL-1β, IL-4, IL-6, IL-8 and IL-10 INF-γ, TNF-α) [15] and allergens [5,16,17], including zearalenone (ZEN), deoxynivalenol (DON) and mixtures thereof [18,19]. 

Mycotoxicosis caused by DON and/or ZEN reduced the number of mucus producing cells and decreased glycocalyx secretion in pigs exposed to low doses of those mycotoxins [20]. Intoxication with higher doses of ZEN alone exerted different effects by stimulating the activity of goblet cells and mucinogen granules [21]. Maresca and Fantini [2] demonstrated that intestinal mucosa is also affected by other mycotoxins which can contribute to inflammatory processes and uncontrolled proliferation of mucosal cells. The mechanisms responsible for mycotoxins’ effects on mucus production have not yet been identified. Trichothecenes and estrogenic hormones were found to inhibit protein synthesis, and ZEN could be included in this group of mycotoxins [2]. 

The effects of ZEN and DON on local enteric immunity have not been investigated to date, but it can be assumed that ZEN is a potential and DON is a definite immunosuppressive agent [2]. Those mycotoxins participate in immunosuppressive processes that lead mainly to intestinal inflammations. Hyperadditive synergistic interactions contribute to the above [22]. Some mycotoxins have direct or indirect pro-inflammatory effects, and they exacerbate the existing inflammations [23]. They can provoke inflammations indirectly by modifying intestinal permeability and contributing to antigen transfer from the intestines, or directly by stimulating the release of pro-inflammatory cytokines from the intestinal epithelium [23]. Expression levels, in particular the expression of NOS-2, should be determined to evaluate the immunomodulative properties of the analyzed mycotoxins. 

The objective of this study was to determine the effect of ZEN, DON and mixtures thereof, administered at low doses to gilts, on the expression levels of NOS-1 and NOS-2 proteins in different sections of the porcine gastrointestinal tract. 

## 2. Materials and Methods

All of the experimental procedures involving animals were carried out in compliance with Polish legal regulations determining the terms and methods for performing experiments on animals (opinion of the Local Ethics Committee for Animal Experimentation No. 88/N of 16 December 2009).

The experiment was conducted at the Department of Veterinary Prevention and Feed Hygiene, Faculty of Veterinary Medicine, University of Warmia and Mazury in Olsztyn, Poland, on 75 clinically healthy gilts with initial body weight of 25 ± 2 kg. The gilts were penned in groups with ad libitum access to water. Administered feed was tested for the presence of mycotoxins: ZEA, α-ZEL and DON. Mycotoxin levels in the diets were estimated by common separation techniques with the use of immunoaffinity columns (Zearala-Test^TM^ Zearalenone Testing System, G1012, VICAM, Watertown, USA and DON-Test^TM^ DON Testing System, VICAM, Watertown, USA) and high performance liquid chromatography (HPLC) (Hewlett Packard, type 1050 and 1100) [24] with fluorescent and/or UV detection techniques. 

The animals were divided into three experimental groups (Z, D and M; *n* = 18 in each group) and one control group (C; *n* = 21). Group Z animals were orally administered ZEN at 40 μg/kg BW, group D animals were orally administered DON at 12 μg/kg BW, and group M animals were orally administered a mixture of ZEN and DON (40 μg ZEN/kg BW + 12 μg DON/kg BW). Group C pigs were fed a placebo. In all experimental groups, mycotoxins were administered at doses below NOAEL values [22]. Both mycotoxins were synthesized and standardized by the Department of Chemistry of the Poznań University of Life Sciences under the supervision of Professor Piotr Goliński. The experiment covered a period of 42 days. Three animals from each experimental group were sacrificed on days 1, 7, 14, 21, 28, 35 and 42 (a total of 12 gilts on each day), excluding day 1 when only three control group animals were scarified. 

### 2.1. Reagents

Analytical samples of the studied mycotoxins were administered *per os* daily in gelatin capsules before the morning feeding. Mycotoxin samples were diluted in 300 μL 96% ethyl alcohol (96% ethyl alcohol, SWW 2442-90, Polskie Odczynniki Chemiczne SA) to obtain the required doses (subject to body weight). The resulting solutions were stored at room temperature for 12 h to evaporate the solvent. The animals were weighed every seven days to update mycotoxin doses for each gilt.

### 2.2. mRNA Isolation and cDNA Synthesis

Tissue samples were collected from the porcine gastrointestinal tract (liver (L)—left lobe, duodenum (DU)—first and middle sections, jejunum (J)—middle section, ascending colon (AC)—middle section, descending colon (DC)—middle section) and rinsed in PBS. Tissue samples were placed in RNAlater® solution (Invitrogen, Carlsbad, California, USA) to stabilize RNA. They were incubated at 4 °C for 24 h and frozen at −80 °C. RNA was isolated from the porcine digestive tract with the use of the Total RNA Mini Plus kit (A&A Biotechnology, Gdynia, Polska). Tissue samples of 200 g were weighed and homogenized in TissueLyser II (Qiagen, USA). Total RNA was extracted in accordance with the manufacturer’s protocol. RNA concentrations and purity were determined in the Nano Vue spectrophotometer (GE Health Care, Buckinghamshire UK). RNA quality was evaluated by electrophoresis in 2% agarose gel. The resulting total RNA was dissolved in an aqueous solution and stored at −80 °C until analysis by reverse transcription PCR (RT-PCR). RT-PCR was performed with the use of Fermentas reagents (Lithuania). The volume of the RNA solution containing 5 μg of total RNA was determined, and it was supplemented to 12.5 μg through the addition of RNase-free water and 1 μg of Oligo(dT)_18_ (0.5 µg) primers (Fermentas, Lithuania). The resulting mixture was incubated at −65 °C for 5 minutes and cooled on ice. 4 μL of 5 × RT buffer, 0.5 μL of 20U RNase inhibitor (RiboLock™ RNase Inhibitor) (Fermentas, Lithuania), 2 μL of dNTP mix, 10 mM each (Fermentas, Lithuania), and 1 μL of 200U reverse transcriptase (RevertAid™ Transcriptase) (Fermentas, Lithuania) were added. The resulting mixture was incubated at 42 °C for 60 min, and the reaction was terminated at 70 °C for 10 min. The reverse transcription reaction was carried out in the Personal Mastercycler Eppendorf thermocycler (Hamburg, Germany). The cDNA synthesis reaction mixture was used in Real-Time PCR.

### 2.3. Real-Time PCR

Real-Time PCR was performed in the Genomics and Transcriptomics Laboratory of the Department of Animal Anatomy, Faculty of Veterinary Medicine of the University of Warmia and Mazury in Olsztyn. Real-Time PCR was carried out to determine the *m*RNA expression levels of NOS-2 and NOS-1 genes in reference to the GAPDH gene. Specific primers for the above genes were designed in the Pick Primer Blast application based on the following sequences: NOS-2–NM_001143690.1, NOS-1–XM_003132898.3 and GAPDH–NM_001206359.1 (Table 1). Reaction tubes were filled with 25 μL of the reaction mix, and the assay was performed in the 7500 Fast Real-Time PCR System thermocycler (Applied Biosystems, Carlsbad, California USA) under the following conditions: initial denaturation −10 min/95 °C, followed by 40 cycles: denaturation −15 s/95 °C, primer annealing −1 min/60 °C. The following reagents were used: 12.5 μL of FastStart Universal SYBR Green Master (Rox) (Roche, Vaud, Switzerland), 10.5 μL of nuclease-free water, 1 μL of cDNA, 1 μL of the primer mix, 5 µM each (forward + reverse). All samples were analyzed in duplicates.

**Table 1:** Real-time PCR primers.

Control gene	Primer sequence (sense and anti-sense)	Primer annealing temperature (°C)	Amplicon length (bp)	Gene bank No.
NOS-1	f: CCATGGCCGCCGATGTCCTC r: CGGTTGTCATCCCTCAGCCTGC	60 °C	109 bp	XM_003132898.3
NOS-2	f: CTCCAGGTGCCCACGGGAAA r: TGGGGATACACTCGCCCGCC	60 °C	117 bp	NM_001143690.1
GAPDH	f: TTCCACCCACGGCAAGTT r: GGCCTTTCCATTGATGACAAG	60 °C	69 bp	NM_001206359.1

### 2.4. Statistical Analysis

The data were grouped based on: (i) the administered mycotoxins relative to experimental dates and (ii) experimental dates relative to the analyzed mycotoxins. Differences between the administered mycotoxins relative to experimental dates and differences between experimental dates relative to the analyzed mycotoxins were analyzed. The results were processed statistically in the Statistica application. Differences between groups (mycotoxin or date) were determined by ANOVA. The equality of group variances was tested by the Brown-Forsythe test. When ANOVA revealed significant differences between groups (*P* < 0.01—highly significant differences, 0.01 < *P* < 0.05—significant differences, *P* > 0.05—no differences), Tukey’s HSD test was used to determine differences between specific groups. 

## 3. Results

**Figure 1:**
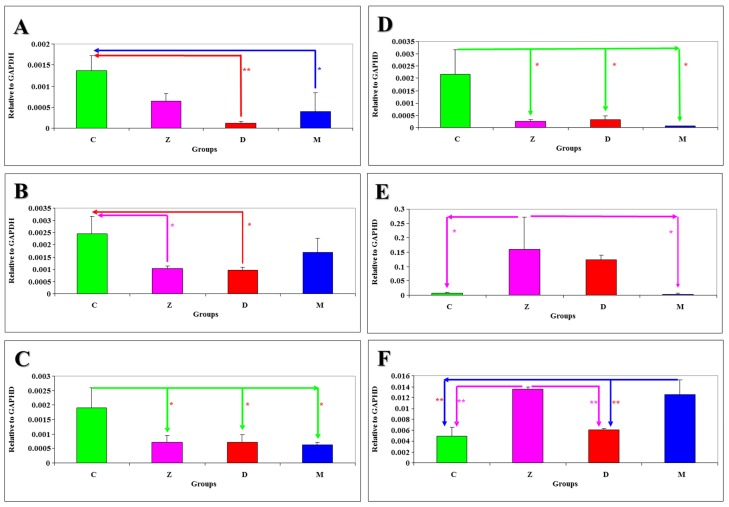
The results of statistical analysis of *m*RNA expression levels of nitric oxide synthase (NOS)-1 and NOS-2 genes in selected sections of the porcine gastrointestinal tract and the liver on different days of the experiment relative to the GAPDH gene are presented in: (**A)** Jejunum NOS-1 (sampling date I); (**B**) Descending colon NOS-1 (sampling date I); (**C**) Descending colon NOS-1 (sampling date V); (**D**) Jejunum NOS-1 (sampling date VI); (**E)** Duodenum NOS-2 (sampling date VI); (**F)** Liver NOS-2 (sampling date I).

The results of statistical analysis of *m*RNA expression levels of NOS-1 genes in selected sections of the porcine gastrointestinal tract and the liver on different days of the experiment relative to the GAPDH gene are presented in Figure 1A–D. Differences at *P* < 0.05 were reported: on sampling date I—between group M and group C in tissue J (Figure 1A), between groups Z and D and group C in tissue DC (Figure 1B); on sampling date V—between group C and the remaining groups in tissue DC (Figure 1C); on sampling date VI—between group C and the remaining groups in tissue J (Figure 1D). Differences at *P* < 0.01 were observed only on date I between group D and group C in tissue J (Figure 1A).

The results of statistical analysis of *m*RNA expression levels of NOS-2 genes in selected sections of the gastrointestinal tract and the liver of gilts intoxicated with mycotoxins on different days of the experiment are presented in Figure 1E–F. Differences at *P* < 0.05 were reported between group Z and groups C and M on date VI in tissue DU (Figure 1E). Differences at *P* < 0.01 were observed on date I between group Z and groups C and D, and between group M and groups C and D in tissue L (Figure 1F).

**Figure 2:**
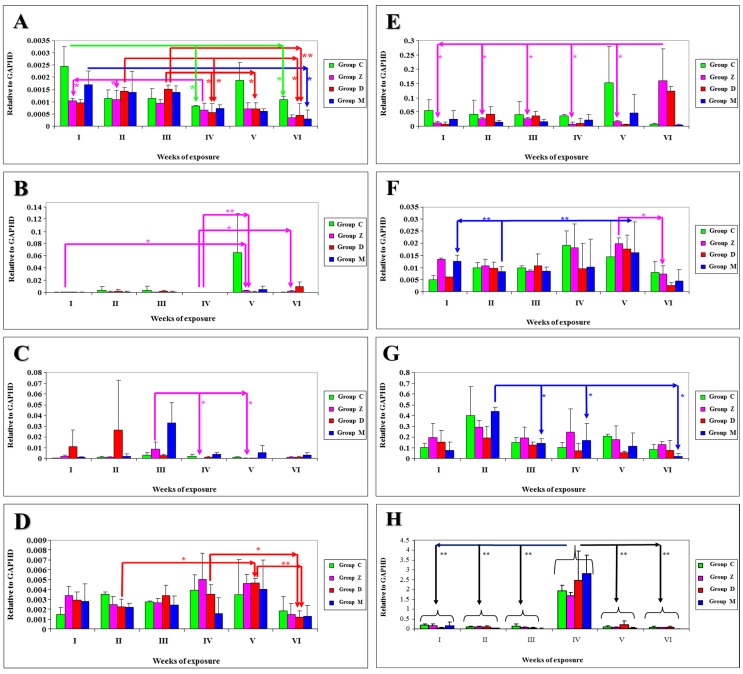
The results of statistical analysis of mRNA expression levels of NOS-1 genes in selected sections of the gastrointestinal tract and the liver of gilts intoxicated with mycotoxins in different groups throughout the entire experiment are presented in: (**A**) Descending colon (NOS-1); (**B**) Duodenum (NOS-1); (**C**) Ascending colon (NOS-1); (**D**) Liver (NOS-1); (**E**) Duodenum (NOS-2); (**F**) Liver (NOS-2); (**G**) Descending colon (NOS-2); (**H**) Jejunum (NOS-2).

The results of statistical analysis of *m*RNA expression levels of NOS-1 genes in selected sections of the gastrointestinal tract and the liver of gilts intoxicated with mycotoxins in different groups throughout the entire experiment are presented in Figure 2A–D. Differences at *P* < 0.05 were reported: in group C in tissue DC between date I and dates IV and VI (Figure 2A); in group Z—in tissue DU between dates I and V and between dates IV and VI (Figure 2B) and in tissue AC between date III and dates IV and V (Figure 2C), in tissue DC between date IV and dates I and II (Figure 2A); in group D—in tissue DC between date III and dates IV and V and between date II and dates IV and VI (Figure 2A), in tissue L between dates II and V and between dates IV and VI (Figure 2D); in group M—in tissue DC between dates I and VI (Figure 2A). Differences at *P* < 0.01 were observed: in group Z—in tissue DU between dates IV and V (Figure 2B); in group D—in tissue DC between dates III and VI (Figure 1A) and in tissue L between dates V and VI (Figure 2D).

The results of statistical analysis of *m*RNA expression levels of NOS-2 genes in selected sections of the gastrointestinal tract and the liver of gilts intoxicated with myxotoxins in different groups throughout the entire experiment are presented in Figure 2E–H. Differences at *P* < 0.05 were reported: in group Z—in tissue DU between date VI and the remaining dates (Figure 2E), in tissue L between dates VI and V (Figure 2F); in group M—in tissue DC between date II and dates III, IV and VI (Figure 2G). Differences at *P* < 0.01 were observed: in all experimental groups in tissue J between date IV and the remaining dates (Figure 2H); in group M—in tissue L between date II and dates I and V (Figure 2F). A high number of differences were verging on the statistically significant level (0.1 > *P* > 0.05), representing statistical tendencies.

The results of statistical analysis of *m*RNA expression levels of NOS-1 and NOS-2 genes in selected sections of the gastrointestinal tract and the liver of gilts intoxicated with mycotoxins on different experimental dates (Figure 1A–F) point to a decreasing trend in the *m*RNA expression levels of NOS-1 genes (Figure 1A–D) in all experimental groups in comparison with group C, between group M and groups Z and D, and between group Z and group D. A similar trend was noted in the *m*RNA expression levels of NOS-2 genes (Figure 1E–F), but an increase in *m*RNA expression was noted in group D in a comparison with group Z. 

The values of NOS-1 gene expression in selected sections of the gastrointestinal tract and the liver of gilts intoxicated with mycotoxins in different groups throughout the experiment (Figure 2A–D) were not uniformly distributed, and the median for selected tissues in different groups was determined at: in group C—0.012336 for DU, 0.001267 for J, 0.001316 for AC, 0.001425 for DC and 0.002838 for L; in group Z—0.001275 for DU, 0.000346 for J, 0.002249 for AC, 0.000795 for DC and 0.003281 for L; in group D—0.002531 for DU, 0.000262 for J, 0.007142 for AC, 0.000940 for DC and 0.002988 for L; in group M—0.001160 for DU, 0.000163 for J, 0.008002 for AC, 0.001026 for DC and 0.002392 for L. The lowest quartile of gene expression values was observed on date VI in 7 cases (3 × in DC and L in groups Z, D and M, and 1× in AC in group C) and on date IV in 6 cases (4 × in DU and 1 × in DC in group C and in AC in group Z). The highest quartile of gene expression values was noted on date V in 6 cases (3 × in DU in groups C, Z and M, 2 × in L in groups D and M, 1 × in J in group D), on date III in 5 cases (4 × in AC and 1 × in DC in group D) and on date I in 4 cases (2 × in J in groups Z and M and 2 × in DC in groups C and M).

The values of NOS-2 gene expression in selected sections of the porcine gastrointestinal tract and the liver across groups throughout the experiment (Figure 2E–H) were not uniformly distributed, and the median for selected tissues in different groups was determined at: in group C—0.055680 for DU, 0.416960 for J, 0.068257 for AC, 0.173322 for DC and 0.011045 for L; in group Z—0.041299 for DU, 0.349643 for J, 0.105470 for AC, 0.204706 for DC and 0.006676 for L; in group D—0.037921 for DU, 0.486018 for J, 0.062965 for AC, 0.109968 for DC and 0.009398 for L; in group M—0.021214 for DU, 0.500869 for J, 0.077001 for AC, 0.158163 for DC and 0.009989 for L. The highest quartile of gene expression values was noted: in DC on date II and in J on date IV in all groups, and in L in groups Z, D and M on date V. The lowest quartile of gene expression values was observed: on date VI in 12 of 20 identified cases (3 × in J and DC in groups C, Z and M; 3 × in L in groups Z, D and M; 2 × in DU in groups C and M, 1 × in AC in group Z); on date V in 4 cases (2 × in AC in groups Z and M, 1 × in DU and 1 × in DC in group D). All median values of *m*RNA expression levels of NOS-2 genes were higher than the median values of NOS-1 expression, excluding DU median values in group D. 

## 4. Discussion

The presence of pathogens in digest can modulate the local immune response. The degree of immunomodulation is determined by the dose of the pathogenic substance, such as a mycotoxin. On the other hand, the clinical picture of intoxication does not reveal such a tendency, which is consistent with the low dose hypothesis proposed by Vandenberg *et al.*, [25]. The induction of regulatory T cells (Treg) is observed. This mechanism is deployed by pathogens, and not only, is to escape the immune response [26], and it observed during chronic and persistent infections. Very little is known about the possible effects of mycotoxins which can be ingested in small doses with feed over prolonged periods of time (monodiet) or on a regular basis.

Reactive oxygen species are activated before Treg induction [14]. They include NO, one of the main signaling molecules in the immune system. NO is active at the place of release or it may be transferred across mucous membranes into surrounding tissues. It is one of the most active smooth muscle relaxants. NO is catalyzed from arginine by NOS. Its activity is strictly determined by its concentrations at the site of the reaction which, in turn, are dependent on the expression levels of specific NOS isomers. At high concentrations, NO exerts proinflammatory and cytotoxic effects directly or indirectly via its active derivatives (nitrite, nitrogen trioxide, peroxynitrite, nitrosoperoxycarbonate). At low, physiological concentrations, NO regulates homeostasis in circulatory, respiratory [27] and immune systems and controls nerve conduction [28,29].

The results of our study investigating the *m*RNA expression levels of NOS-1 and NOS-2 genes in gilts administered very low doses of individual mycotoxins (ZEN and DON) or the mycotoxin mix for 42 days are partially consistent with published data. Significant differences (at *P* < 0.05 and *P* < 0.01) were observed in the *m*RNA expression levels of NOS-1 genes in selected sections of the porcine gastrointestinal tract and the liver on different days of the experiment (Figure 1A–F), in particular in tissues J and DC on dates I and VI. A decreasing trend in median values was reported in comparison with group C and between groups administered DON. A similar trend was observed in the *m*RNA expression levels of NOS-2 genes, but the noted values were significantly higher in comparison with NOS-1 expression. A drop in the *m*RNA expression levels of both genes was observed in groups Z, D and M. This suggests that low doses of ZEN, DON or the mycotoxin mix in feed (below NOAEL values) inhibit both NOS isomers, which probably lowers NO synthesis. The results shown in Figure 1 could suggest that ZEN was the inhibitory factor at the initial stage of the experiment, but the longer the exposure to ZEN, the decrease in NOS-1 mRNA expression was leveled out, whereas the drop in NOS-2 mRNA expression was more pronounced in group Z.

Similar statistical tendencies were noted with respect to the *m*RNA expression levels of NOS-1 and NOS-2 genes in selected sections of the gastrointestinal tract and the liver of gilts intoxicated with mycotoxins in different groups throughout the entire experiment (Figure 2E–H). A decreasing trend was intensified over time and in groups administered the mycotoxin/mycotoxins. The only exceptions were tissue sections where significant differences were observed, mostly NOS-2 expression values in tissues DU, J, AC and DC. 

Our findings indicate that both mycotoxins inhibit the *m*RNA expression of genes controlling constitutive isomer NOS-1 and inducible isomer NOS-2. It can be expected that low doses of ZEN or DON administered with feed (below NOAEL value) over prolonged periods of time can modify gastrointestinal functions due to a decrease in the concentrations of NO which inhibits NANC transmitters [30,31]. Low levels of NO can probably speed up peristalsis in the esophagus, stomach and intestines, inhibit gastric accommodation responses (receptive and adaptive relaxation, antral contraction) and increase anal sphincter pressure, thus slowing down gastric emptying and digesta passage through the intestines [6,32]. 

In consequence, decreased *m*RNA expression of the NOS-1 gene, released in the enteric nervous system, should slow down intestinal peristalsis and sphincter function. A drop in the *m*RNA expression of the NOS-2 gene should decrease intestinal permeability and inhibit bowel secretion [33]. The above conclusions were formulated by directly extrapolating from the symptoms caused by high mycotoxin doses to those caused by low doses. However, recent research findings provide evidence contrary to this assumption (low dose hypothesis). The influence of very low doses (below NOAEL values) of any hormonal agent which modulates tissue activity could be different. Undesirable hormonally active substances [25] such as ZEN, which can act as a signaling molecule, could serve as an example.

Interestingly, a decrease in the *m*RNA expression of genes controlling both NOS isomerases was particularly noted in distal sections of the digestive tract. The explanations can be provided for the above observation. Firstly, mycotoxins have antibacterial properties, and they decrease the populations of pathogenic microorganisms which are one of the key pro-inflammatory agents and which stimulate NO production [34]. Secondly, when administered at low doses, the analyzed mycotoxins inhibit the *m*RNA expression of both NOS genes, which could have therapeutic implications [31,35] at high levels but not at physiological levels of NO. The presence of low doses of ZEN and DON (below NOAEL value) in feed inhibits inflammatory processes in the digestive tract, in particular in tissues J, AC and DC. The above could be attributed to the “escape” of signaling molecules from local and systemic immune activation, similar to that observed when Tregs are induced by bacterial pathogens [26] in chronic infections.

## References

[1] Xu X., Xu P., Ma C., Tang J., Zhang X. (2013). Gut microbiota, host health, and polysaccharides. Biotechnol. Adv..

[2] Maresca M., Fantini J. (2010). Some food-associated mycotoxins as potential risk factors in humans predisposed to chronic intestinal inflammatory diseases. Toxicon.

[3] Pinton P., Guzylack L., Kolf-Clauw M., Oswald I.P. (2012). Effects of some fungal toxins, the trichothecenes, on the epithelial intestinal barrier. Curr. Immunol. Rev..

[4] Groschwitz K.R., Hogan S.P. (2009). Intestinal barrier function: Molecular regulation and disease pathogenesis. J. Allergy Clin. Immunol..

[5] Lechner M., Lirk P., Rieder J. (2005). Inducible nitric oxide synthase (iNOS) in tumor biology: The two sides of the same coin. Semin. Cancer Biol..

[6] Castro M., Muńoz J.M., Arruebo M.P., Murillo M.D., Arnal C., Bonafonte J.I., Plaza M.A. (2012). Involvement of neuronal nitric oxide synthase (nNOS) in the regulation of migrating motor complex (MMC) in sheep. Vet. J..

[7] Sessa W.C. (1994). The nitric oxide synthase family of proteins. J. Vasc. Res..

[8] Dusting G.J. (1995). Nitric oxide in cardiovascular disorders. J. Vasc. Res..

[9] Arnal J.F., Dinh-Xuanb A.T., Pueyoc M., Darbladea B., Ramia J. (1999). Endothelium-derived nitric oxide and vascular physiology and pathology. Cell Mol. Life Sci..

[10] Bielewicz J., Kurzepa J., Łagowska-Lenard M., Bartosik-Psujek H. (2010). The novel views on the patomechanism of ischemic stroke. Wiad. Lek..

[11] Losada A.P., Bermúdez R., Faílde L.D., Quiroga M.I. (2012). Quantitative and qualitative evaluation of iNOS expression in turbot (Psetta maxima) infected with Enteromyxum scophthalmi. Fish Shellfish Immun..

[12] Mehl M., Daiber A., Herold S., Shoun H., Ullrich V. (1999). Peroxynitrite reaction with heme proteins. Nitric Oxide.

[13] Stuehr D.J., Santolini J., Wang Z.Q., Wei C.C., Adak S. (2004). Update on mechanism and catalytic regulation in the NO synthases. J. Biol. Chem..

[14] Ługowski M., Saczko J., Kulbacka J., Banaś T. (2011). Reactive oxygen and nitrogen species. Pol. Merk. Lek..

[15] Alderton W.K., Cooper C.E., Knowles R.G. (2001). Nitric oxide synthases: Structure, function and inhibiton. Biochem. J..

[16] Sokołowska M., Włodek L. (2001). Good and bad sides of nitric oxide. Folia Cardiol..

[17] Tokuhara K., Hamada Y., Tanaka H., Yamada M., Ozaki T., Matsui K., Kamiyama Y., Nishizawa M., Ito S., Okumura T. (2008). Rebamipide, anti-gastric ulcer drug, up-regulates the induction of iNOS in proinflammatory cytokine-stimulated hepatocytes. Nitric Oxide.

[18] Gajęcki M., Gajęcka M., Jakimiuk E., Zielonka Ł. (2005). Feedingstuffs and human health. Pol. J. Food. Nutr. Sci..

[19] Zielonka Ł., Obremski K., Gajęcka M., Rybarczyk L., Jakimiuk E., Gajęcki M. (2010). An evaluation of selected indicators of immune response in pigs fed a diet containing deoxynivalenol, T-2 toxin and zearalenone. Bull. Vet. Inst. Pulawy.

[20] Obremski K., Zielonka Ł., Gajęcka M., Jakimiuk E., Bakuła T., Baranowski M., Gajęcki M. (2008). Histological estimation of the small intestine wall after administration of feed containing deoxynivalenol, T-2 toxin and zearalenone in the pig. Pol. J. Vet. Sci..

[21] Obremski K., Gajęcka M., Zielonka Ł., Jakimiuk E., Gajęcki M. (2005). Morphology and ultrastructure of small intestine mucosa in gilts with zearalenone mycotoxicosis. Pol. J. Vet. Sci..

[22] Boermans H.J., Leung M.C.K. (2007). Mycotoxins and the pet food industry: Toxicological evidence and risk assessment. Int. J. Food. Microbiol..

[23] Maresca M., Yahi N., Younès-Sakr L., Boyron M., Caporiccio B., Fantini J. (2008). Both direct and indirect effects account for the proinflammatory activity of enteropathogenic mycotoxins on the human intestinal epithelium: Stimulation of interleukin-8 secretion, potentiation of interleukin-1beta effect and increase in the transepithelial passage of commensal bacteria. Toxicol. Appl. Pharmacol..

[24] Obremski K., Gajęcki M., Zwierzchowski W., Bakuła T., Apoznański J., Wojciechowski J. (2003). The level of zearalenone and α-zearalenol in the blood of gilts after feeding them of feed with a low content of zearalenone. J. Ani. Feed Sci..

[25] Vandenberg L.N., Colborn T., Hayes T.B., Heindel J.J., Jacobs D.R., Lee D.-H., Shioda T., Soto A.M., vom Saal F.S., Welshons W.V. (2012). Hormones and endocrine-disrupting chemicals: Low-dose effects and nonmonotonic dose responses. Endocr. Rev..

[26] Silva-Campa E., Mata-Haro V., Mateu E., Hernández J. (2012). Porcine reproductive and respiratory syndrome virus induces CD4^+^CD8^+^CD25^+^Foxp3^+^ regulatory T cells (Tregs). Virology.

[27] Ziętkowski Z., Ziętkowska E., Bodzenta-Łukaszyk A. (2009). Exhaled nitric oxide measurements in the diagnosis of respiratory diseases. Alerg. Astma Immun..

[28] Nussler A.K., Billiar T.R. (1993). Inflammation, immunoregulation and inducible nitric oxide synthase. J. Leukoc. Biol..

[29] Kroncke K.D., Fehsel K., Kolb-Bachofen W. (1995). Inducible nitric oxide synthase and its product nitric oxide, a small molecule with complex biological activities. Biol. Chem..

[30] Gupta A., Sharma A.C. (2004). Despite minimal hemodynamic alterations endotoxemia modulates NOS and p38-MAPK phosphorylation via metalloendopeptidases. Mol. Cell Biochem..

[31] Grześk E., Grześk G., Koziński M., Stolarek W., Zieliński M., Kubica J. (2011). Nitric oxide as a cause and a potential place therapeutic intervention in hyporesponsiveness vascular in early sepsis. Folia Cardiol..

[32] Bennett M.R. (1997). Non-adrenergic non-cholinergic (NANC) transmission to smooth muscle: 35 years on. Prog. Neurobiol..

[33] Dijkstra G., van Goor H., Jansen P.L., Moshage H. (2004). Targeting nitric oxide in the gastrointestinal tract. Curr. Opin. Invest. Drugs.

[34] Davila A.-M., Blachier F., Gotteland M., Andriamihaja M., Benetti P.-H., Sanz Y., Tomé D. (2013). Re-print of “Intestinal luminal nitrogen metabolism: Role of the gut microbiota and consequences for the host”. Pharmacol. Res..

[35] Yang E.J., Yim E.Y., Song G., Kim G.O., Hyun C.G. (2009). Inhibition of nitric oxide production in lipopolysaccharide-activated RAW 264.7 macrophages by Jeju plant extracts. Interdisc. Toxicol..

